# Understanding the factors of wearable devices among the patients with thyroid cancer: A modified UTAUT2 model

**DOI:** 10.1371/journal.pone.0305944

**Published:** 2024-07-26

**Authors:** LingLi Song, BinXian Li, HaiBo Wu, CuiCui Wu, XueQi Zhang

**Affiliations:** 1 Department of Clinical Laboratory, Associated Hospital, Beihua University, Jilin, China; 2 Department of Nuclear Medicine, Associated Hospital, Beihua University, Jilin, China; Shandong University, CHINA

## Abstract

Wearable devices hold promising prospects on a global scale, including in China. Thyroid cancer prevalence is notably high in China.This purpose of this researchwas to provide an updated theoretical model for assessing Chinese thyroid cancer patients’ intentions towards wearable devices, based on the UTAUT2 framework, and to ascertain the factors that have an impact on these intents. A cross-sectional study with an institutional focus wasconducted from January 20, 2023, to June 30, 2023, at several general hospitals in China. Five hundred participants were recruited to identify predictors of wearable device use.The questionnaire survey about patients’ intentionswas collected using a face-to-face method, employing a random sampling technique for patient selection. Four hundred sixty-nine individuals (93.8%) had the intention to use wearable devices. The intentions were highly impacted by performance expectancy (PE), effort expectancy (EE), social influence (SI), hedonic motivation (HM), price value (PV) and habit (HA). Usage intention (UI)was a statistically predictor of Usage behavior (UB). The facilitating condition(FC) was not significant. Gender positively moderated the relationship between EE and UI. Income positively moderated the relationship between all variables and UI.Overall, the utilization of wearable devices among patients diagnosed with thyroid cancer has demonstrated considerable potential. This study offers a series of suggestions for digital health developers,healthcare decision-makers,doctors and patients.

## Introduction

Thyroid cancer (TC) stands as the most prevalent neoplastic disorder affecting the endocrine system. Among these, the incidence of papillary thyroid cancer (PTC) is the highest [1, 2]. The incidence of TC keeps rising worldwide. The year 2020 witnessed the emergence of 570,000 new case [3, 4]. An estimated total of 220,000 new cases of TC were reported in Mainland China in 2020 [5, 6]. At the same time, resources of China for thyroid cancer healthy management are low [7]. Wearable devices, as an emerging technology, serve as a means of self-health management.Wearable devices are systematically classified into four primary uses, namely: (1) monitoring health and safety parameters, (2) managing chronic illnesses, (3) diagnosing and providing therapeutic treatments for diseases, (4) facilitating rehabilitation efforts [8].The application and acceptance of wearable devices are widely studied in many medical fields, such as the use of wearable devices by patients with chronic diseases like hypertension and diabetes for self-management of health [9, 10].

Wearable devices can gauge cancer patients’ health. The outcomes include some fundamental health data, behavioral patterns, and early inflammatory signs. Integrating this data may improve cancer treatment [8, 11].Wearable devices in healthcare are gaining popularity. An essential consideration in employing wearables in the realm of cancer pertains to the acceptance and adherence of patients who are already grappling with the challenges of a debilitating illness and undergoing oncological therapy.Wearable devices have been developed and applied in health monitoring of cancer patients and assisted rehabilitation after treatmentand there are reported studies [12]. This includes applications in thyroid diseases as well [13, 14]. The use of wearable devices in oncology provides significant additional value during every phase of treatment. Through the additional value they provide, doctors may enhance patient care by providing valuable information on optimizing physical health management at various phases. For example, they can advise patients to engage in appropriate exercise, adjust their sleep patterns, and intervene in their lifestyle to effectively guide patients in self-management of their health [15]. However, the application of wearable devices in oncology lacks clear standards [16–18]. Cancer patients form a distinct demographic,and their adoption of wearable technology is often contingent upon their medical requirements and the explicit endorsements of physicians, so potentially disregarding the emotional and subjective experiences of patients. Research reports are limited [19–22]. Therefore, this study’s approach focuses on the patient’s perspective, combining the unified theory of acceptance and use of technology model (UTAUT2) to investigate the factors influencing the use of wearable devices.

The aim of this research is to provide an updated theoretical framework for assessing Chinese thyroid cancer patients’ intentions towards wearable devices, based on the UTAUT2 framework. Moreover, this research is to ascertain the factors that have an impact on these intents. This study has the potential to provide significant knowledge to healthcare professionals and researchers, enabling them to more accurately identify patients who might benefit from using wearable devices. This work contributed to the advancement and integration of wearable devices in China, specifically for the objective of Self-care management in individuals with thyroid cancer. The implications of the research have broad implications for several areas, including practice, policy, and future research efforts. These areas are focused on reducing thyroid cancer burden and its com- plications in China by using digital health interventions. This study has significant value as it provides useful feedback for normal practices. Extensive literature review has identified a lack of research on the intentions of individuals with thyroid cancer to use wearable devices, for monitoring thyroid cancer-related vital signs [23–25]. So, it is crucial to evaluate the degree of intention among prospective users before the implementation of wearable medical devices.

## Materials and methods

### Background

Several models of technological acceptance have been utilized to study the adoption of information systems, including the diffusion of innovation theory, enhanced technology acceptance model and UTAUT, among others [26]. Therefore, it is crucial to carefully choose a suitable theory and model as the foundation for understanding user behavior concerning the technologies. This research presents a theoretical model rooted in UTAUT2, which has been shown to be useful in explaining wearable device adoption, to address the research inquiries [27]. The UTAUT model was established by a thorough analysis of eight distinct technology acceptance models, with the aim of presenting a consolidated perspective on the adoption of technology [28]. In accordance with this conceptual, it is hypothesized that the intention to utilize a novel technology is impacted by four core constructs: performance expectancy (PE),effort expectancy (EE), social influence (SI) and facilitating condition (FC).Expanding upon the existing framework, Venkatesh et al. Enhanced the UTAUT model by incorporating three supplementary factors: hedonic motivation (HM),price value(PV) and habit(HA) [29].According to previous research findings, the UTAUT2 model can explain approximately 72% of usage intention (UI) and around 52% of usage behavior (UB) [30]. The UTAUT2 framework comprises a essential core framework that allows investigators to add external variables relevant to the specific technology being studied (such as wearable devices within the healthcare domain)and deepen the model’s scope by quantifying their influence on user interface[26].

The paper specifically chooses thyroid cancer patients as the study participants from the broader population of cancer patients due to three distinct reasons. Firstly, thyroid cancer patients often have a favorable prognosis after surgery, with a high likelihood of survival. Nevertheless, the need for extended health monitoring necessitates the individual to take personal responsibility for managing their own health. Secondly, indicators for monitoring thyroid cancer are representative, because the illness often affects parameters such as heart rate, blood pressure and blood sugar levels. Thirdly, thyroid cancer exhibits a substantial prevalence in northern locations. Additionally, patients often do not need radiation or chemotherapy after treatment, hence having little influence on their fundamental health condition. Hence, the study participants exhibit a wide range of diversity [31].

The field of oncology has yet to fully explore the potential of wearable devices. The UTAUT2 framework’s constructs have been meticulously included within the scope of this research project in light of these factors.

### Research model

**Fig 1:**
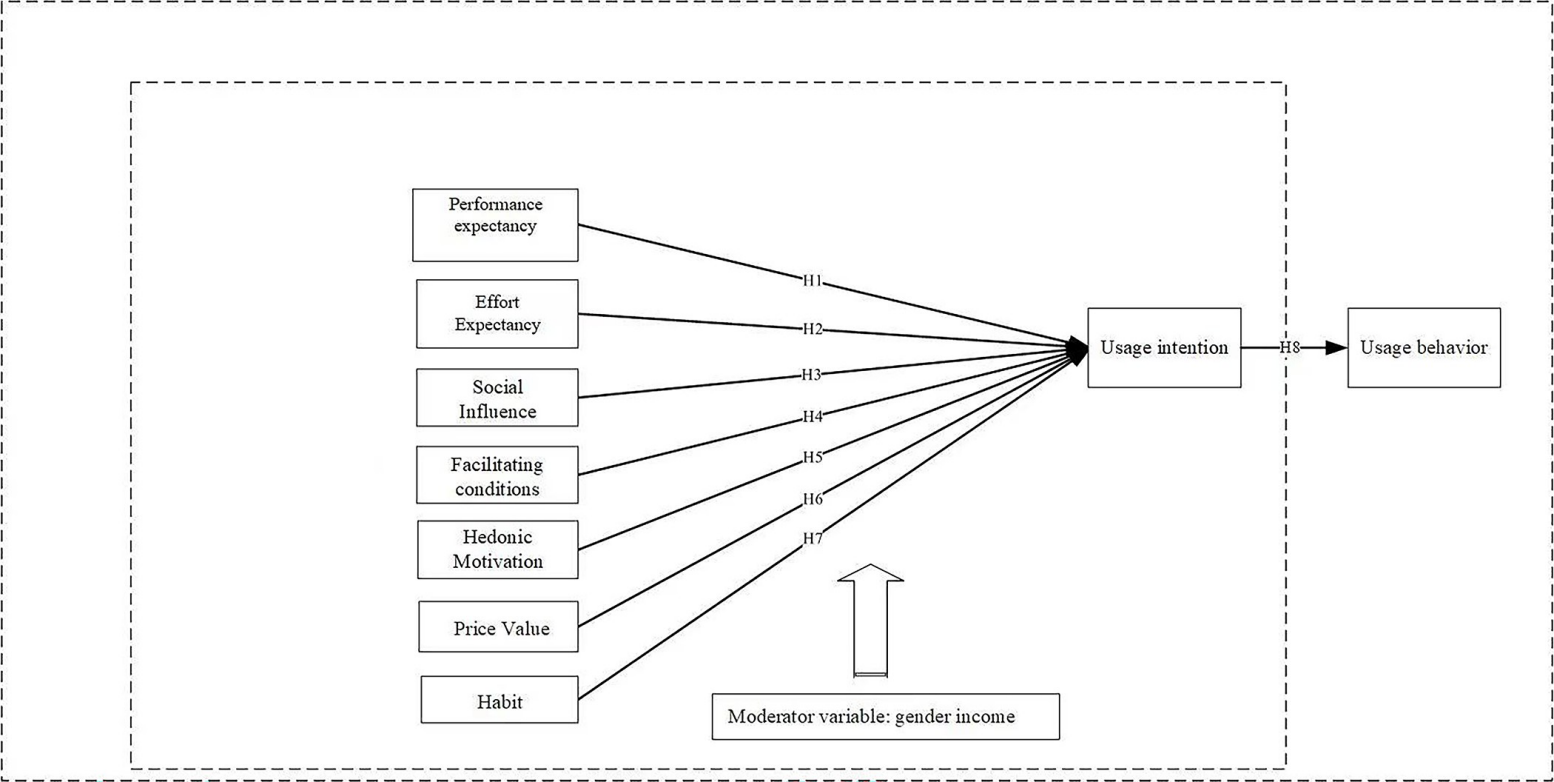
Research model.

We build an empirically verified model based on Venkatesh’s UTAUT2 framework and relevant prior studies. The aim is to understand the behavioral intention and influencing factors of cancer patients’ use of wearable devices. The model consists of three parts. Firstly, the UTAUT2 model contains seven exogenous variables, namely PE, EE, SI, FC, HM, PV, HA. Secondly, the model includes the endogenous variable UI and the dependent variable UB [29]. Thirdly, to investigate whether gender and income influence the predictive factors of patients’ adoption of wearable devices, they are introduced into the model as moderating variables [32–37]. The research model that is being offered is provided in Fig 1.To facilitate better comprehension, the revised concepts and proposed hypotheses will be further elaborated in the following subsections.

PE is defined as the extent to which an individual anticipates that using a specific technology can augment overall performance [29]. In this research, PE refers to the ability of patients using wearable devices to perceive that they can enhance their ability to manage their own health and better engage in healthcare activities.Empirical evidence has shown the significance of PE as a predictive factor for UI in the context of using diverse technologies, including wearable devices [38, 39].The significant impact of PE on the willingness to adoptmHealth services in Bangladesh [40].Thus,the hypothesis was posited:

H1.Performance expectancy increases thyroid cancer patients’ wearable health device usage intention.

The concept of effort expectation pertains to a person’s cognition regarding the minimal effort required when employing an advanced technology [29]. In this study, EE represents the degree to which patients find it easy or difficult to use wearable devices. Prior studies have provided evidence indicating that users’ inclination to embrace mHealth technology exhibits an upward trend when the technology is designed with user-friendly features. It may be inferred that a person’s perception of a wearable device’s ease of use may affect their likelihood of actually using it. The present research on wearable devices adoption shows a favorable correlation between EE and both UI and UB [38, 41].Thus, the hypothesis was posited:

H2. Effort expectancy increases thyroid cancer patients’ wearable health device usage intention.

SI can be defined as the degree to which an individual believes influential persons expect them to utilize a specific technology [29]. The importance of social factors on individuals’ inclination to embrace technology has been widely acknowledged in numerous theories of technology adoption [42]. However, certain research, particularly those involving professions with high levels of autonomy like doctors, showed a non-significant impact [43]. Social impact includes family, friends, and society. In this study, tumor patients may value and be influenced more by their doctors’ opinions. Thus, the hypothesis was posited:

H3. Social influence increases thyroid cancer patients’ wearable health device usage intention.

FC encompass an individual’s views regarding the necessary resources and assistance required for the successful execution of a certain behavior [29]. In this study, FC refers to the resources, support and expertise accessible to patients for using wearable devices. Several studies indicate that people are more inclined to adopt new technologies when there are better favorable conditions to facilitate their use[38, 44]. Additionally, wearable devices need on network transmission to monitor data, making FC relatively important for UI [45]. Thus, the hypothesis was posited:

H4.Facilitating condition increases thyroid cancer patients’ wearable health device usage intention.

HM refers to the sensation of enjoyment or pleasure derived from the utilization of new technology. It significantly affects technology acceptance and utilization [29]. In this study, we operationalize the construction of HM by incorporating pleasure, enjoyment and psychological safety through questionnaires. Patients may experience pleasure and enjoyment from using wearable devices, as well as psychological satisfaction from achieving health goals. Many research investigations have stressed the relevance of HM in wearable devices usage [46]. However, research in developing countries like Malaysia suggests that HM may not significantly affect health wearable technology adoption [47, 48]. To validate the impact of HM, the hypothesis was posited:

H5. Hedonic motivation increases thyroid cancer patients’ wearable health device usage intention.

The PV pertains to an individual’s assessment of the balance between the advantages provided by a technology and its corresponding monetary expenditure [29]. Significantly, Previous research has indicated that the intention to adopt a new technology is influenced by the PV [46]. Wearable technologies have potential advantages, but many are prohibitively expensive for low-income people. A Saudi Arabian research revealed that PV did not have an impact on the behavioral intention to utilize wearable technologies [49]. Thus, the hypothesis was posited:

H6.Price value increases thyroid cancer patients’ wearable health device usage intention.

Habit can be defined as the extent to which individuals display behaviors in an automatic manner as a result of previous learning [29]. In this research, habit is defined as the inherent behavioral patterns that are developed by patients via regular use of wearable devices. It can be constructed through the frequency of use, daily routines, automaticity and repetitiveness of patients. Some empirical studies have confirmed the correlation between HA and UI and revealed underlying mechanisms[26, 46]. However, a Dutch study found that HA negatively affected people’s willingness to utilize health monitoring wearable devices [50]. Thus, the hypothesis was posited:

H7. Habit increases thyroid cancer patients’ wearable health device usage intention.

The correlation between UI and actual UB has been extensively studied in various research fields, demonstrating the reliability of UI as a predictive factor for actual UB [51, 52]. The empirical examination has provided evidence to support the efficacy of the behavioral intention construct in explaining a user’s actual utilization of technology [29, 44]. The research found that doctors’ actual use behavior (UB) strongly impacts their behavioral intention to adopt mobile medical technology [53]. The inclusion of this construct facilitates the revelation of its contributing function to the behavior of real use and its impact on improving the status of living among cancer patients who utilize wearable devices. Thus, the hypothesis was posited:

H8.usage intention increases thyroid cancer patients’ wearable health device usage behavior.

Gender has demonstrated its significance as a robust predictor of consumer intention and behavior in numerous social science disciplines [54]. Thyroid cancer (TC) has a notable gender disparity, primarily affecting females with a prevalence three to four times greater than males. However, males tend to have a more severe form of the disease [37]. Papillary thyroid cancer (PTC) constituted 90% of all thyroid cancerand the incidence ratio of women to men for small PTC was 4.39:1 between 2013 and 2017 [36].Based on predictive study, the gender disparity in the incidence rate of thyroid cancer will continue until 2032, with a male-to-female ratio reaching up to 3.0 [55]. Therefore, when formulating management plans for thyroid nodules, patient sex should be taken into account to attain the ultimate objective of reducing morbidity and improving outcomes [56]. Consequently, it is reasonable to choose gender as a moderator. Investigating the various influences originating from gender becomes a interesting thing for further research [54]. In light of the aforementioned evidence, the following hypotheses can be drawn:

H9.Gender positively moderates performance expectancy on thyroid cancer patients’ wearable devices usage.

H10. Gender positively moderates effort expectancy on thyroid cancer patients’ wearable devices usage.
H11.Gender positively moderates social influence on thyroid cancer patients’ wearable devices usage.
H12. Gender positively moderates hedonic motivations on thyroid cancer patients’ wearable devices usage.
H13. Gender positively moderates price value on thyroid cancer patients’ wearable devices usage.
H14. Gender positively moderates habit on thyroid cancer patients’ wearable devices usage.

The moderating effect of income on willingness to adopt wearable devices belongs to the research gap and has not been reported. Nevertheless, income has been acknowledged as a robust predictor of consumer intention and behavior. An empirical investigation demonstrates that wearable health monitors have the capacity to strengthen healthcare systems in areas characterized by low- and middle-income status [57]. The presence of income disparities has significant ramifications in the realm of thyroid cancer screening, including several factors such as incidence rates, postoperative complications, and mortality rates. This phenomenon is particularly evident in the setting of Korea [35]. Furthermore, it should be noted that the prevalence of thyroid cancer is not limited exclusively to areas characterized by greater socioeconomic status, but rather encompasses low- and middle-income nations as well [58]. Income has been identified as a standalone prognostic factor for individuals diagnosed with stage IV anaplastic Thyroid Cancer [59]. Moreover, variations in income are also evident in environmental exposure among older persons, thereby underscoring the discrepancies that exist between individuals belonging to lower-and higher-income groups [32]. In light of the aforementioned evidence, the following hypotheses can be drawn:

H15.Income positively moderates performance expectancy on thyroid cancer patients’ wearable devices usage.
H16. Income positively moderates effort expectancy on thyroid cancer patients’ wearable devices usage.
H17. Income positively moderates social influence on thyroid cancer patients’ wearable devices usage.
H18. Income positively moderates hedonic motivations on thyroid cancer patients’ wearable devices usage.
H19. Income positively moderates price value on thyroid cancer patients’ wearable devices usage.
H20. Income positively moderates habit on thyroid cancer patients’ wearable devices usage.

### Study design and participants

This study with a focus on hospitals was conducted from January 20, 2023, to June 30, 2023, in Northern China. These hospitals are all comprehensive institutions affiliated with universities. Diagnosis, treatment, prognosis, and follow-up for thyroid cancer were accessible at these general hospitals. All adult thyroid cancer patients at the aforementioned hospitals were included in the source population. During the specified time of data collection, the research sample included adult individuals diagnosed with thyroid cancer who sought medical treatment in both outpatient and inpatient settings. The research excluded children under 18, as surveying them involves substantial logistical preparation and financial resources. Children’s cognitive ability in judging new technologies may also skew outcomes. Those with severe illness and communication problems were excluded from the research.

### Sampling procedure and sample size

The selection of research participants originated from the general hospitals. Data from the target population were gathered using a non-probability convenience sampling approach, with proportional allocations made for each hospital. The monthly participation in the questionnaire was determined by the combined number of patients from both inpatient and outpatient settings.

Before commencing data collection, we conducted exploratory factor analysis on a sample of 113 participants. The results indicated that Cronbach’s alpha coefficient for each latent variable exceeded 0.7, signifying high reliability. Following the preliminary steps of language analysis, conversion, and necessary adjustments, the actual investigation commenced. Out of a total of 500 participants, 469 expressed their willingness to use wearable devices. Thanks to the conduction of in-person interviews and the provision of prompt explanations and communications, no invalid questionnaires were encountered. Responses such as a score of 0, failure to answer questions entirely, or incomplete questionnaire responses were deemed inadequate.

### Data collection

This study employed a standardized questionnaire based on Venkatesh’s work and other UTAUT2 model investigations. The data about patients’ intention was collected using a face-to-face survey. The questionnaire comprised three parts. Firstly, the study examines socio-demographic characteristics using a set of seven items. Secondly part explores clinical issues using a set of three items. Finally, this part consists of 20 affirmative hypotheses representing the predictive factors in the model. The questionnaire consists of a total of 36 items, evaluating components through a five-point Likert scale. The scale ranges from 1 to 5.

### Data analysis

The software applications employed in the process of data processing and model evaluation included Statistical Package for Social Science (SPSS) and Analysis of Moment Structures (AMOS). SPSS 25 was utilized for analyzing demographic characteristics, clinically relevant variables, and patients’ intentions. This study employed the structural equation modeling (SEM) analysis framework proposed in prior research. Model analysis and evaluation relied on AMOS 26.

### Ethics

Ethics clearance was granted by the Affiliated Hospital of Beihua University, registration number 2023–47. Researchers obtained written informed consent from all individuals. Data were collected anonymously to protect participant privacy. The ethical principles of the Helsinki Declaration were followed in this study.

## Results

### Clinical characteristics of thyroid cancer patients

We investigated 500 participants. The survey found that 469 (93.8%) individuals expressed an intent to use wearable devices. Respondents had an average age of 39 years old, with 366 people (78.0%) falling between the ages of 30 and 50.Out of the total respondents, 350 individuals (74.6%) were female. The majority of patients, 371 (79.1%), were married. Non-religious participants constituted 331 (70.6%) of the total. In terms of occupation, 223 (47.5%) people worked in government. A total of 395 (84.2%) individuals had a higher education degree or above. The survey found that 269 participants (57.4%) had yearly per capita incomes over 100,000 RMB in China (Table 1). Most research participants, 459 (97.9%), had papillary thyroid cancer. About 433 (92.7%) of research participants took medicine regularly, and 348 (74.2%) had no comorbidities (Table 2).

**Table 1:** Participants socio-demographic parameters in the research.

item	Category	Frequency(N)	Percentage(%)
Gender	Male Female	248 221	52.9 47.1
Age(year)	18–30 30–50 >50	65 366 38	13.9 78.0 8.1
Educational level	Uneducation Education Higher education	20 54 395	4.3 11.5 84.2
Marital status	Single Married Separated widowed	42 371 38 18	9.0 79.1 8.1 3.8
Religion	Yes No	138 331	29.4 70.6
Occupation	Government Employee self-employed Unemployed	223 102 55 89	47.5 21.7 11.7 19.1
Income	>150000RMB 100000-150000RMB 50000-100000RMB <50000RMB	36 207 203 23	7.7 44.1 43.3 4.9

**Table 2:** Features of the research participants.

Clinical classifications	Category	Number(N)	Percentage(%)
thyroid cancer type	PTC Others	459 10	97.9 2.1
Treatment regularly	Yes No	433 36	92.3 7.7
Comorbidity	Yes No	121 348	25.8 74.2

### Measurement model

The adequacy of the fit of the measurement model is assessed via Confirmatory Factor Analysis (CFA). Composite reliability, convergent validity, and discriminant validity of the measurement model are evaluated.The factor loading for each item in the study varied from 0.66 to 0.92. All factor loadings exceeded the threshold of 0.6, indicating the high reliability of the study’s questions [60, 61]. CFA of the measurement model demonstrated a good fit, meeting the specified criteria (Table 3). Evaluation of reliability and validity are critical. If the measurement model fails to meet the validity and reliability cut-off values, subsequent path analysis would lose its meaningfulness.Tables 4 and 5 present the measurement model evaluation findings. The composite reliability of the model ranged from 0.79 (UB) to 0.93 (SI). Composite Reliability(CR)which has an acceptable value of 0.7 or above, were used to assess the construct dependability. These models have superior composite reliability measurements, with CR values over 0.7. The AVE of the model range from 0.56 (usage behavior) to 0.75 (usage intention). AVE which has an acceptable value of 0.5 or above, were used to assess the convergent validity. Convergent validity was attained for the model (Table 4). According to the Farnell-Larcker criterion, discriminant validity is established when items load more heavily on their assigned construct than on other constructs, and the square root of the average variance extracted of a construct is greater [62]. For every construct—from 0.818 for performance expectation to 0.840 for PV—the square root of the AVE (diagonal values) exceeded the highest correlation with any other construct. Discriminant validity was attained for the model(Table 5).

**Table 3:** Model fit indices.

Fit indices	Threshold value	Results obtained	Conclusion
Chi-square/degree of freedom	< = 3	1.77	Accepted
Goodness-of-fit-index(GFI)	>0.9	0.90	Accepted
Adjusted goodness-of-fit-index(AGFI)	>0.8	0.88	Accepted
Comparative fit index(CFI)	>0.9	0.96	Accepted
Root means square error of approximation(RMSEA)	<0.08	0.04	Accepted
Standardized root mean squared residual (SRMR)	<0.08	0.03	Accepted

**Table 4:** Composite reliability and convergent validity.

Construct	Indicators/Items	Standard factor loading	CR	AVE
Performance expectancy	PE1 PE2 PE3 PE4	0.84 0.90 0.83 0.82	0.92	0.72
Effort expectancy	EE1 EE2 EE3 EE4	0.78 0.81 0.80 0.88	0.89	0.67
Social influence	SI1 SI2 SI3 SI4	0.83 0.85 0.89 0.88	0.93	0.74
Facilitating condition	FC1 FC2 FC3 FC4	0.86 0.81 0.92 0.82	0.92	0.74
Hedonic motivation	HM1 HM2 HM3 HM4	0.76 0.83 0.71 0.84	0.84	0.61
Price Value	PV1 PV2 PV3 PV4	0.75 0.88 0.85 0.79	0.89	0.67
Habit	HA1 HA2 HA3 HA4	0.88 0.91 0.83 0.84	0.92	0.73
Usage Intention	UI1 UI2 UI3 UI4	0.86 0.83 0.84 0.83	0.84	0.75
Usage Behavior	UB1 UB2 UB3 UB4	0.72 0.86 0.72 0.66	0.79	0.56

**Table 5:** Discriminant validity.

Construct	PE	EE	SI	PC	HM	PV	HA	UI	UB
PE	0.818								
EE	0.287	0.833							
SI	0.271	0.365	0.838						
FC	0.542	0.617	0.355	0.831					
HM	0.555	0.548	0.323	0.694	0.826				
PV	0.106	0.188	0.136	0.194	0.108	0.840			
HA	0.136	0.193	0.118	0.196	0.136	0.437	0.836		
UI	0.156	0.201	0.174	0.246	0.159	0.455	0.424	0.831	
UB	0.161	0.157	0.114	0.191	0.131	0.406	0.410	0.492	0.826

### Structural model

This research checked collinearity to ensure no strong correlations between constructs. The results show that the values of variance inflation factor (VIF) are all below the threshold of 3.0,showing that this study has no multicollinearity (Table 6).

**Table 6:** Multicollinearity test.

Exogenous Construct	Variance Inflation Factor
PE	1.192
EE	1.379
SI	1.367
FC	1.159
HM	1.031
PV	1.613
HA	1.161

### Hypothesis verification

**Fig 2:**
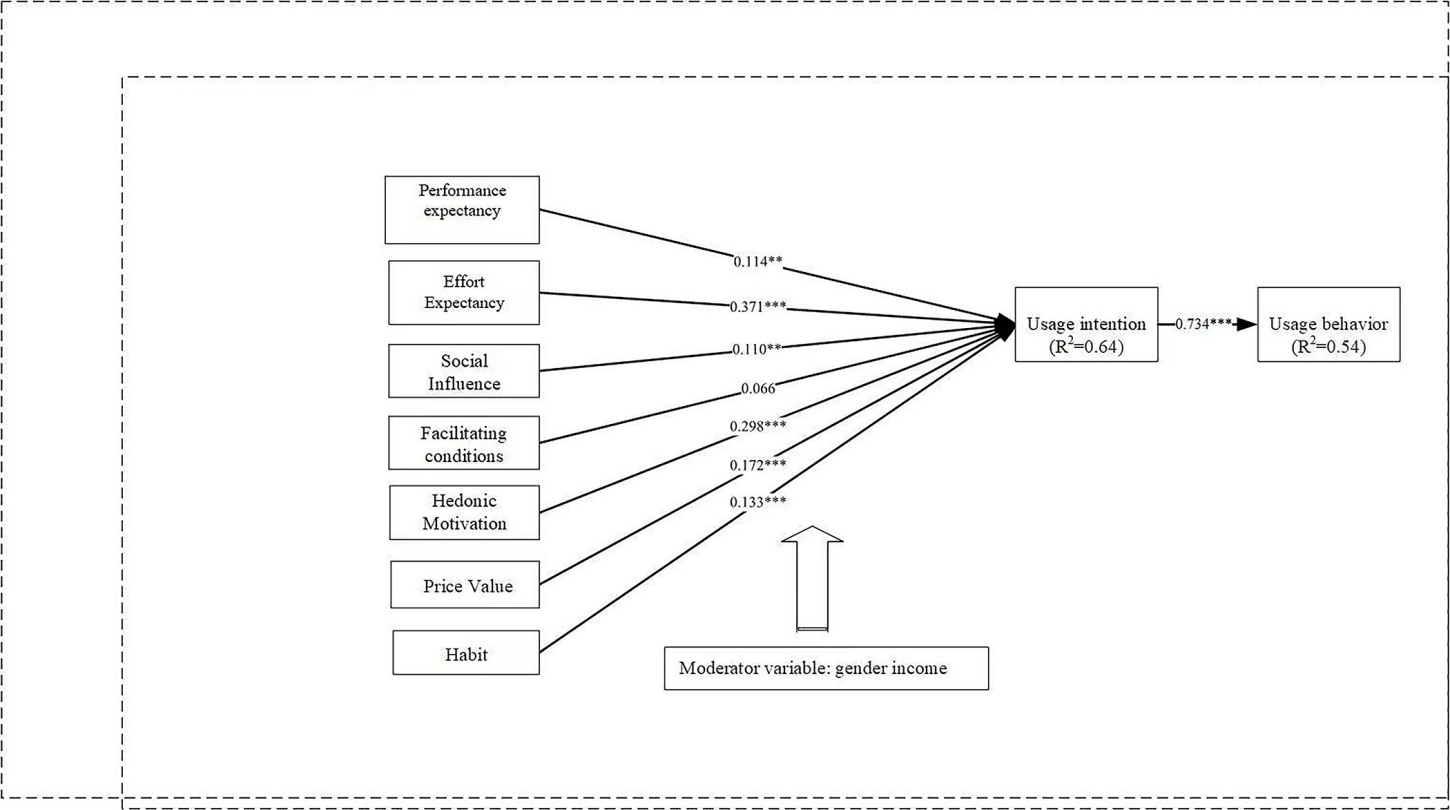
Research model path analysis.*R*^*2*^ is the coefficient of determination.

The model was analyzed and path coefficients verified after validating its applicability. P-values showed that H4 was rejected, while the other 7 pathways were statistically significant. The seven external components together explained 64.0% of the endogenous construct, specifically referred to as usage intention (UI). The R2 value for UI was 0.64. The impact of UI on UB was 54% and R2 value for UB was 0.54.This finding showed that the proposed model predicted accurately. As shown in Fig 2.

The research analyzes the relationships between the external components and endogenous construct using path coefficients (β), critical ratio, standard error and p-value. The significance level used in this inquiry is 0.05. As a result, theories with a p-value greater than 0.05 are considered to be not statistically significant [63–65]. We enumerate the theories and conclusions drawn from the analytical process. There are seven connections that have significance.(Table 7)

**Table 7:** Analysis of factors of intention.

Path	Estimate	β	Standard error	Critical ratio	P-Value	Result
PE-UI	0.142	0.114	0.048	2.978	**	Supported
EE-UI	0.444	0.371	0.055	8.026	***	Supported
SI-UI	0.114	0.110	0.044	2.597	**	Supported
FC-UI	0.073	0.066	0.041	1.765	0.078	Not Supported
HM-UI	0.386	0.298	0.050	7.712	***	Supported
PV-UI	0.209	0.172	0.059	3.530	***	Supported
HA-UI	0.148	0.133	0.042	3.532	***	Supported
UI-UB	0.443	0.734	0.033	13.348	***	Supported

Note

* *p*< 0.05

** *p*< 0.01

*** *p*< 0.001

### Moderation effects

The objective of this section was to investigate how gender and income moderate the complicated interaction between PE, EE, SI, HA, HM and PV on wearable device intentions. Two sets of model comparisons were conducted, including unconstrained and constrained (structural weight) models, to assess the influence of moderators. If the observed difference between the two models reached statistical significance(p-value<0.05), it indicated that the hypothetical moderator variable had been validated as a moderator [66]. Table 8 shows that gender positively affected the UI based on EE, with a bigger effect on male participants than female ones. Thus, H10 is supported. Table 9 shows that income positively moderated the impact of all hypotheses on wearable device intention. Participants with incomes above 100,000 CNY had a greater association between each variable and intention.

**Table 8:** Moderating effects of gender.

Path	Moderator Gender	Path coefficient	P-value	Model test		Result
				ΔX2	P-value	
PE-UI	Male Female	0.108 0.132	0.024 0.018	0.020	0.887	Not Supported
EE-UI	Male Female	0.416 0.336	<0.001 <0.001	3.957	0.046	Supported
SI-UI	Male Female	0.122 0.116	0.018 0.043	0.041	0.839	Not Supported
HM-UI	Male Female	0.301 0.290	<0.001 <0.001	1.623	0.203	Not Supported
PV-UI	Male Female	0.193 0.154	<0.001 <0.001	1.079	0.299	Not Supported
HA-UI	Male Female	0.181 0.087	<0.001 0.134	1.669	0.196	Not Supported

**Table 9:** Moderating effect of income.

Path	Moderator Gender	Path coefficient	P-value	Model test		Result
				ΔX2	P-value	
PE-UI	<100000 >100000	0.000 0.179	0.999 <0.001	8.166	0.004	Supported
EE-UI	<100000 >100000	0.348 0.458	<0.001 <0.001	6.292	0.012	Supported
SI-UI	<100000 >100000	0.088 0.219	0.064 <0.001	9.080	0.003	Supported
HM-UI	<100000 >100000	0.220 0.334	0.003 <0.001	4.262	0.039	Supported
PV-UI	<100000 >100000	0.018 0.289	0.821 <0.001	13.893	<0.001	Supported
HA-UI	<100000 >100000	0.010 0.193	0.887 <0.001	8.297	0.001	Supported

## Discussion

### Intentions in patients

The rising global prevalence of cancer and the increased focus on improving the quality of life for cancer patients will drive the market for wearable gadgets specifically designed for this population.

Without understanding the willingness of this population to use such devices, designers will be unable to provide substantive assistance to tumor patients. The literature published in 2022 represents the first assessment of compliance to wearable medical devices among the oncology patients [8]. Nevertheless, there is a lack of adequate study on the variables that influence the adoption of wearable technologies.The current investigation introduced and experimentally examined a theoretical framework that has potential in predicting the adoption of wearable devices among individuals diagnosed with thyroid cancer in Northern China.The research found 469 patients intended to adopt wearable devices (93.8%). The results of our investigation demonstrate that nearly 90 percent of participants exhibited a preference for wearable devices. The survey’s findings indicate a significant inclination towards the use of wearable devices surpassing those of other groups examined in Canada and the United States [28, 67, 68]. Possibly based on two reasons. Firstly, this survey was conducted face to face,which patients believed could better protect their privacy.This approach also provides patients with a more detailed explanation of their intentions,clarifying the function of wearable devices, thus enabling patients to better understand the significance of the survey.Secondly, cancer patients, compared to healthy populations, tend to consider the severity of the disease. Out of concern for their health, participants express a strong interest in newly developed technologies that are beneficial for maintaining their health.

### Factors influencing wearable adoption

This study has made some contributions to increasing the willingness of diagnosed thyroid cancer patients to adopt wearable devices, and it also holds significant implications for future research.Regarding the driving factors for the adoption of wearable devices, current research has mainly focused on chronic disease and healthy populations.The main focus of this research is on individuals who have been diagnosed with thyroid cancer.

Our study found substantial correlations between the willingness to adopt wearable devices and factors such as PE, EE, SI, HM, PVand HA.Except for FC, all were demonstrated to be predictive factors for the adoption of wearable devices.This result may be properly explained by taking into account the peculiar traits of cancer patients, who make up a distinct group that values need above convenience.The findings demonstrate that using intention positively influenced UB.

We found that EE(β = 0.444, P< 0.001) and HM(β = 0.386, P<0.001)are the most important driving factors for the adoption of wearable devices. It is worth discussing that EE is consistent with previous literature reports [38, 44, 69, 70], while HM contradicts previous reports [71, 72]. Possible explanations for this discrepancy may include the following two points.Firstly, variations in the characteristics of the individuals being studied may explain this phenomenon. Cancer patients encounter a greater number of physiological and psychological obstacles and often devote a greater amount of time and energy to their healthcare compared to persons who are in good health.Secondly, it’s possible that contemporary individuals have become more familiar with information technology, and they have opportunities to learn about new technologies through social media [42, 73]. Therefore, if novel technology can provide more pleasure and simplicity of use, it will promote wider adoption within this group of individuals.

We found that PV(β = 0.209, P<0.001),PE(β = 0.142, P < 0.003),SI(β = 0.114, P = 0.009), and HA(β = 0.148, P<0.001) have relatively similar effects as determining variables. Firstly, the PV study’s findings are consistent with those of past research on wearable devices adoption [29, 46]. The observed phenomenon may perhaps be ascribed to the financial resources available to households of individuals diagnosed with cancer. The aforementioned claim is substantiated by a study conducted in South Korea, which presents persuasive data regarding the influence of wealth disparities on many facets of thyroid cancer, encompassing screening, incidence rates, surgical complications, and fatality rates [35, 74]. Secondly, the PE study’s findings are consistent with those of past research on wearable devices adoption [68, 75, 76].This shows that people utilize wearable devices more when these technologies can better monitor their health and fulfill healthcare tasks faster.The potential explanation for this phenomenon might be ascribed to the recognition of the use of electronic medical devices, such as blood pressure and heart rate monitors. One additional factor might be that cancer patients consider their disease to be severe and believe that new technology is essential to controlling it at home.Thirdly, the SI study’s finding is consistence with those of past research on wearable devices adoption. However, past research also has indicated that people prefer family and friend support over WD for self-management [6, 39, 40, 50, 77]. Additionally, the participants expressed that engaging in face-to-face consultations with healthcare professionals offers a more precise depiction of their illness compared to managing it through the use of wearable health devices. The patients demonstrated greater adherence to the doctor’s recommendations [40].Lastly, the HA study’s finding is consistent with research from South Africa, the United States, and Canada [29, 68, 72]. This demonstrates that users are more likely to want to utilize wearable devices when utilizing them becomes second nature to them and they grow hooked to doing so.The majority of thyroid cancer patients are acquainted with long-term testing methods like monitoring hormone levels and other information technologies. Participants in the research who lived in cities and had experience with these technologies made up the great majority of the group. People could thus believe that utilizing comparable technology regularly increases their likelihood of being able to utilize wearable health equipment without difficulty.

### Moderation by gender and income

Based on the results of this research, gender was found to play a significant moderating role in the relationship between EE and the inclination to use wearable devices, exhibiting a positive effect. According to the findings of this research, male participants exhibited higher EE and expressed more usage intentions compared to female. This revelation is supported by consistent data from a research carried out in the United States and consistency with the original theoretical model. There seems to be a disparity in the level of awareness of mobile health services between men and women, maybe attributed to men’s greater exposure and familiarity with digital health technology. Men think it’s easier to use wearable health gadgets now that this has happened [78, 79].

This study found that income positively moderated the impact of all hypotheses on wearable health device intention. There is a clear discrepancy seen between households with higher and lower income levels in terms of their inclination to use wearable devices. The aforementioned finding highlights the significance of personal income in shaping the adoption of wearable devices. This research is notable for its innovative incorporation of income as a moderating variable, a dimension that has not been previously investigated within medical frame works. Compared to other research, the disparity in income levels has a noticeable influence on several facets of thyroid cancer, including screening, occurrence, complications after surgery, and death [33, 35]. Furthermore, research has demonstrated that affluence significantly influences how emergent technologies are adopted [32, 57]. Therefore, we chose income as a moderator for our study. One possible reason is that persons with lower income have less contact with emerging technologies. On the other hand, those with higher income levels are more inclined to demonstrate a better level of ease and understanding when it comes to the benefits offered by wearable devices. This may be ascribed to their increased exposure to similar technology.

## Implication

### Theoretical implication

Regarding the acceptance of wearable devices among cancer patients, we constructed the UTAUT2 model and used gender and income as moderating variables. At the same time, establishing a strong basis for subsequent research by academic researchers in the field.The findings of our analysis may provide valuable insights to the academic community on the aspects that have a substantial impact on enhancing UI. The willingness of cancer users can guide market development strategies for enterprise developers and inform the formulation of administrative policies by medical government officials. In addition, this study contributes in the following two aspects. Firstly,the findings demonstrate that the model has a high level of explanatory capability in predicting user acceptability.Additionally, the introduced moderating variables also demonstrate their moderating capabilities. Therefore, future empirical research can adopt this perspective within the healthcare system to examine the willingness to use other new technologies and methodologies. Secondly, there is limited research attention on individuals with cancer, which constitute a special group and represent an insufficiently explored area of wearable gadget advancement. Our research contributes to the current limited knowledge of tumor populations.This fills a new gap in population research on wearable device applications. The techniques described in this research are ideal for evaluating the adoption of technology among individuals with cancer, offering a suitable framework. The influence of EE may operate as a fundamental futureinvestigations.

### Particular implication

There are several practical implications from the findings. First, from the perspective of technical developers, considering that EE, HM and PV are relatively important factors influencing the adoption of wearable devices, wearable devices designed for cancer patients should pay more attention to usability and bring a sense of pleasure to the patients. For example, adding feedback mechanisms and incorporating patient satisfaction and pleasure as evaluation metrics in the development process could be considered. Increasing pleasure is also a pretty straightforward way to enhance the health of cancer patients, which may have something to do with their psychological well-being.Furthermore, considering that gender and income have moderating effects, more humane products could be designed. For example, distinguishing different functions for male and female patients, adding voice input and music functions, offering products with price gradients, and customizing input for monitoring indicators could be implemented.These innovations may assist wearable device development businesses in sustaining their long-term competitiveness. Second, from the perspective of medical personnel, given that research has found SI, HA and PE to impact the adoption of wearable devices, it’s crucial to recognize the significance of medical personnel, especially for cancer patients. If medical personnel advocate for the adoption of a particular technology, patients are likely to perceive it as important. Moreover, the patients’ acceptability will be enhanced if family members aid them in getting acquainted with and cultivating a routine of using wearable devices.Last, from the perspective of the government and the healthcare system, wearable devices offer the ability to comprehensively capture patients’ health data. Governments can develop intelligent systems for collecting and storing health data, allowing doctors to conveniently and thoroughly understand patient information. This can lead to improved clinical treatment efficiency and service quality. Efficiently mitigating the existing problem of insufficient medical resources, streamlining healthcare services, and optimizing the allocation of medical resources.

## Study limitation and future research

First, the research design was non-experimental,primarily focusing on studying the influencing factors of patients’ adoption of wearable devices.If future research includes the genuine experiential feedback from patients, it may provide an abundance of valuable research discoveries. For example, qualitative exploration of patients using wearable devicesdemonstrates a proactive approach to advancing knowledge in the field. Exploring the nuanced experiences of cancer patients with technology could yield valuable insights into acceptance and intention to use, informing future interventions and research endeavors.

Second, the research sample exclusively consisted of individuals diagnosed with thyroid cancer, all of whom exhibited a singular tumor type. The initial selection of patients with thyroid cancer as research subjects was based on their favorable prognosis and high survival rate. Subsequently, future research can examine more individuals with different tumor types to enhance the study’s generalizability.

Additionally, while this paper delves into multiple factors and draws meaningful conclusions, it must be acknowledged that there are still some limitations. This study concentrated on the original independent variables, eliminating exogenous influences. Next research may include privacy apprehensions, trust, and digital health literacy to the UTAUT2 framework to address these shortcomings. This suggested update addresses existing and future constraints that may affect wearable devices use.
